# Initiation, response assessment, and switch of antibody therapies in patients with severe asthma – A survey among German specialists

**DOI:** 10.1016/j.waojou.2023.100844

**Published:** 2023-11-15

**Authors:** Hendrik Suhling, Dirk Skowasch, Karl-Christian Bergmann, Carlo Mümmler, Roland Buhl, Rainer Ehmann, Eckard Hamelmann, Marco Idzko, Margret Jandl, Christian Schulz, Olaf Schmidt, Christian Taube, Stephanie Korn, Katrin Milger, the GAN registry group

**Affiliations:** a Department of Respiratory Medicine and Infectious Diseases, Hannover Medical School, Germany; b Department of Internal Medicine II - Pneumology/Cardiology, University Hospital Bonn, Bonn, Germany; c Institute of Allergology, Charité – Universitätsmedizin Berlin, Corporate Member of Freie Universität Berlin and Humboldt-Universität zu Berlin, Berlin, Germany; d Department of Internal Medicine V, Ludwig-Maximilians-University of Munich (LMU), Munich, Germany; e Comprehensive Pneumology Center (CPC-M), Helmholtz Center Munich, Member of the German Center for Lung Research (DZL), Munich, Germany; f Pulmonary Department, Mainz University Hospital, Mainz, Germany; g Ambulante Pneumologie Stuttgart, Stuttgart, Germany; h Klinik für Kinder- und Jugendmedizin Kinderzentrum Bethel, Bielefeld, Germany; i Department of Respiratory Medicine, Medical University of Vienna, Vienna, Austria; j Hamburger Institut für Therapieforschung GmbH, Hamburg, Germany; k Bereich Pneumologie Klinik und Poliklinik für Innere Medizin II, University Hospital Regensburg, Regensburg, Germany; l Pneumologische Gemeinschaftspraxis und Studienzentrum KPPK, Koblenz, Germany; m Department of Pulmonary Medicine, University Medical Center Essen-Ruhrlandklinik, Essen, Germany; n IKF Pneumologie Mainz and Thoraxklinik Heidelberg, Mainz and Heidelberg, Germany

**Keywords:** Anti asthmatic drugs, Antibodies, Omalizumab, Mepolizumab, Benralizumab, Dupilumab, Questionnaire, Asthma, Germany

## Abstract

**Background:**

For therapy of severe asthma 5 monoclonal antibodies have been available in Germany up to November 2022, but no clear rules exist on choice of initial therapy, assessment of response, and switch.

**Objective:**

To assess current practice on all aspects of biologic therapy by specialists in Germany.

**Methods:**

A questionnaire was created by specialists for severe asthma, which was tested and modified by further experts. We invited 119 pulmonologists of the German Asthma Net (GAN) to complete the survey and used SoSci Survey and SPSS for data collection and analysis.

**Results:**

Forty-seven pulmonologists took part in the survey with a median annual number of patients treated with biologics of 35, 55% worked in an outpatient practice, and 40% in a hospital. Exacerbations and oral steroid use were the most important factors for the decision to start a biologic therapy. Accordingly, these parameters were also the most relevant for assessment of response. Most participants considered type-2 inflammation biomarkers and comorbidities (foremost CRSwNP and AD) for choosing initial biologic. Asthma Control Test (ACT) was the most common instrument for assessing status of disease control. There was no consensus on thresholds for response of pulmonary function tests including FEV1, FVC, and RV. Eighty-five percent of participants distinguished between “responders”, “partial responders” and “non-responders”. Comorbidities played an important role for the decision to switch to another biologic, eg, when initial therapy had insufficient effectiveness on CRSwNP.

**Conclusion:**

This study provides a detailed insight into current opinions and practice of biologic use in severe asthma in Germany.

## Introduction

Advances in therapy of severe asthma have been made with the introduction of monoclonal antibodies in the last decades. When asthma control is not achieved with high-dose inhaled corticosteroids and bronchodilators, the current Global Initiative for Asthma (GINA) document clearly recommends to favor add-on therapy with antibodies over oral corticosteroids.^1^ GINA suggests to phenotype severe asthma for choice of targeted therapy, but clear recommendations, when to use which antibody, are still weak. At time of the study, 5 different antibodies were available in Germany (omalizumab, mepolizumab, benralizumab, reslizumab, and dupilumab), tezepelumab was later introduced and thus not included.^2^ Omalizumab is indicated for allergic, mepolizumab, benralizumab, and reslizumab for eosinophilic, and dupilumab for type-2 high phenotype.^3^ As these phenotypes overlap in many patients there is more than 1 option for antibody selection. After initiation of a therapy, different proposals on assessment of response have been made, but so far, there is no concrete advice on when and how to change antibody therapy. Recently, a SHARP initiative used a questionnaire for evaluation of continuation criteria, switching, combination, and evaluation of corticosteroid toxicity.^4^

In our present study, we used an online questionnaire to investigate the status quo of antibody handling in severe asthma in Germany covering the 3 topics initiation with choice of antibody, evaluation of response, and potential switch from one to another antibody.

## Materials and methods

**Fig. 1:**
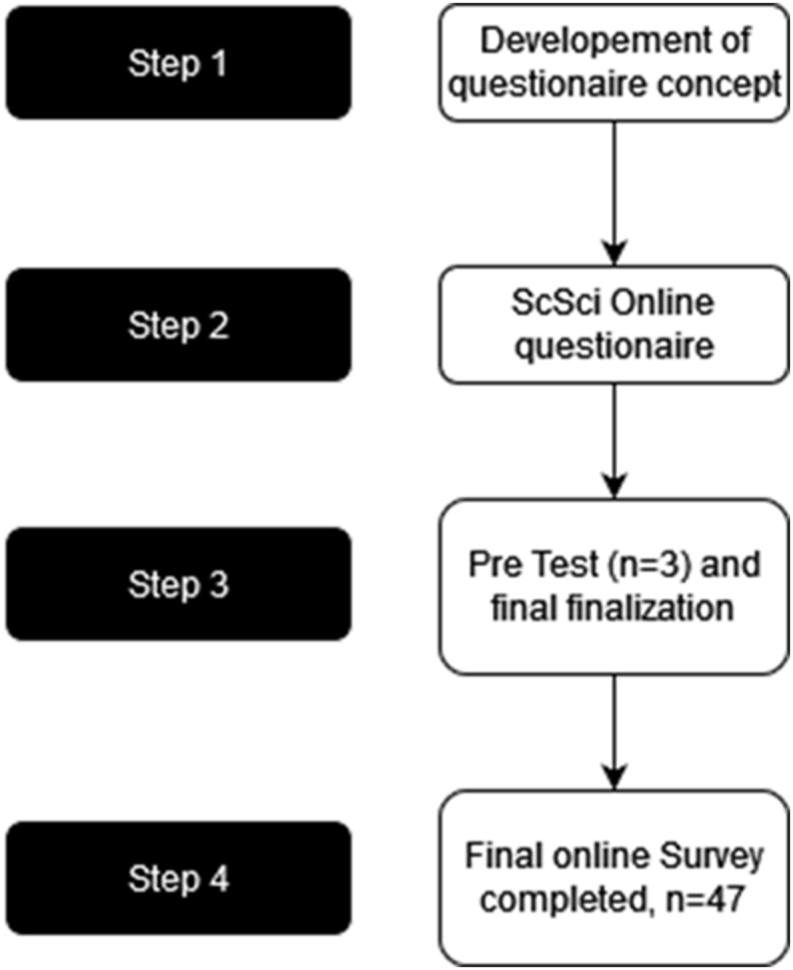
Flow chart of study procedures

The project was started in May 2022 within the German Asthma Net (GAN). GAN is a non-profit organization promoting care for and research on severe asthma and hosting a registry for severe asthma. Currently 119 specialists for severe asthma from centers in Germany are members in GAN. Firstly, the questions for the 3 categories (antibody start/response/switch) were developed and subsequently reviewed within the author team. Further, an online questionnaire using SoSci Survey (Munich, Germany) was created and tested by additional experts for severe asthma with modifications suggested by the experts. Finally, 119 experts from the GAN, all pulmonologists from Germany, were asked via email to answer the 66-item questionnaire anonymously (see supplement) from July 20, 2022 to September 25, 2022. Forty-seven pulmonologists completed the survey. The answers of all participants were equally weighted. Fig. 1 shows the flow-chart of study procedures. Descriptive data analysis was performed using SPSS 28 (IBM). All antibodies investigated here are covered by the statutory health insurance in Germany for the indication of severe asthma when licensing criteria for the specific biologic are fulfilled (omalizumab: severe allergic asthma with sensitization to a perennial allergen; mepolizumab: severe eosinophilic asthma characterized by increased blood eosinophils ≥150/μl, reslizumab: severe eosinophilic asthma characterized by increased blood eosinophils ≥400/μl, benralizumab: severe eosinophilic asthma characterized by increased blood eosinophils ≥300/μl or ≥ 150/μl with ongoing oral corticosteroid (OCS) therapy, dupilumab: severe asthma with type-2 inflammation characterized by increased blood eosinophils ≥150/μl and/or increased FeNO ≥25 ppb). Tezepelumab was not investigated here because it became available in Germany in November 2022, when the data acquisition was already finished.

## Results

Forty-seven pulmonologists from GAN centers in Germany completed the survey. They had a median age of 52 (IQR 47; 60) years and 19 (40%) worked in hospitals with outpatient unit, 26 (55%) in outpatient practice, and 2 in other units. Six participants were pediatric pulmonologists, the others treated adult patients only. In median, participants had 20.5 years of experience in treating patients with asthma. The median annual count of patients under biologic therapy was 35 (IQR 17.5 and 110).

### Initiation and choice of biologic therapy

Before starting an antibody therapy, the most important parameters for identification of severe and uncontrolled asthma were exacerbations and oral-corticosteroid use, followed by hospitalizations, lung function, and an established triple inhalation regime. Over 90% of participants recommended high dose inhaled corticosteroid/long-acting beta agonist (ICS/LABA) therapy and 76% additional long-acting muscarinic antagonists (LAMA) usage before considering start of antibody therapy. Seventy percent thought that a previous or ongoing oral steroid therapy is not a prerequisite for antibody initiation. Missed days at school/work were of minor interest as parameters before therapy.

**Fig. 2:**
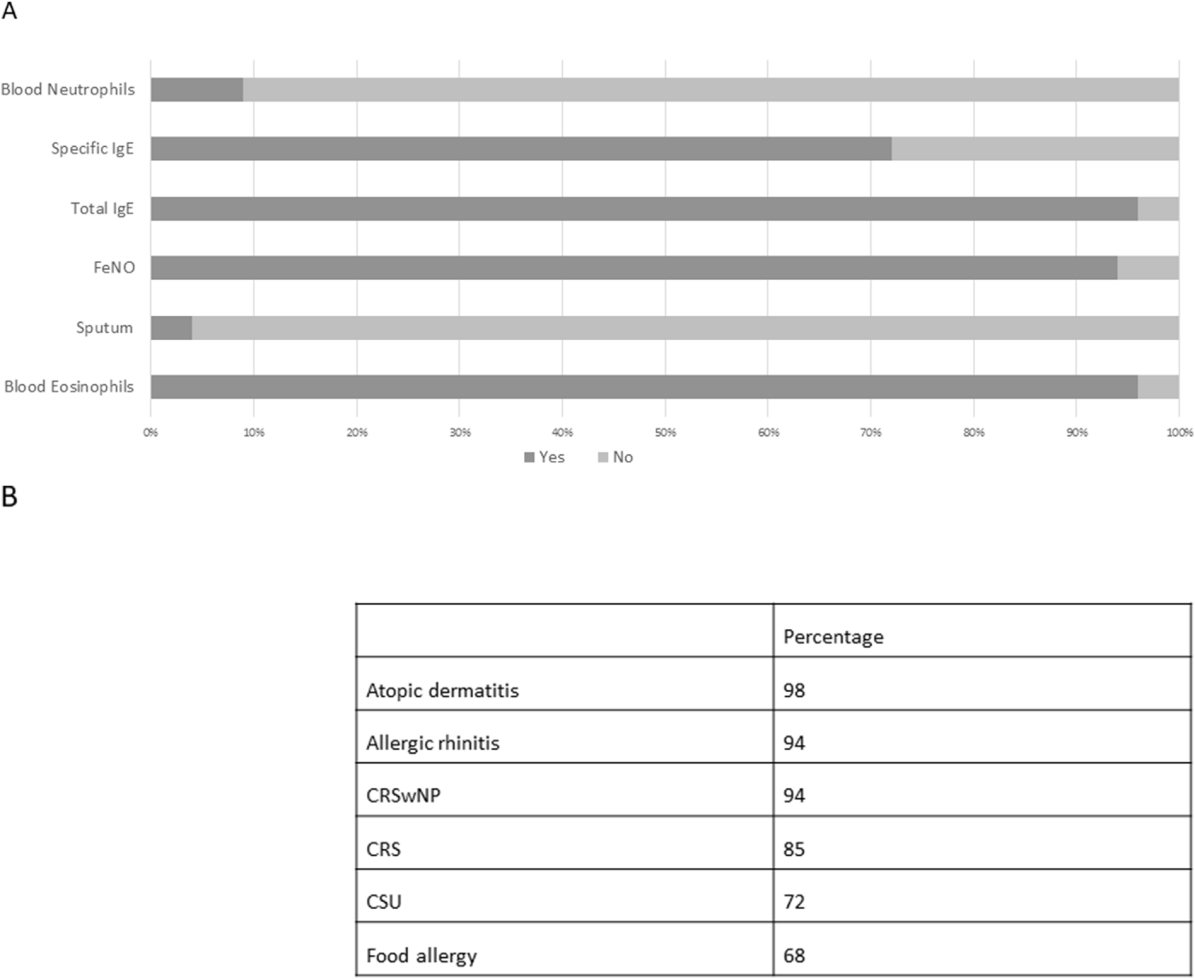
Percentages of specialists performing routine assessment before biologic therapy. A Biomarker. B Relevant *comorbidities* for biologic choice

At the time the survey was answered by the large majority of participants tezepelumab was not yet available in Germany. For choosing an antibody, 96% of doctors took into account the biomarker profile and comorbidities (see Fig. 2).

**Fig. 3:**
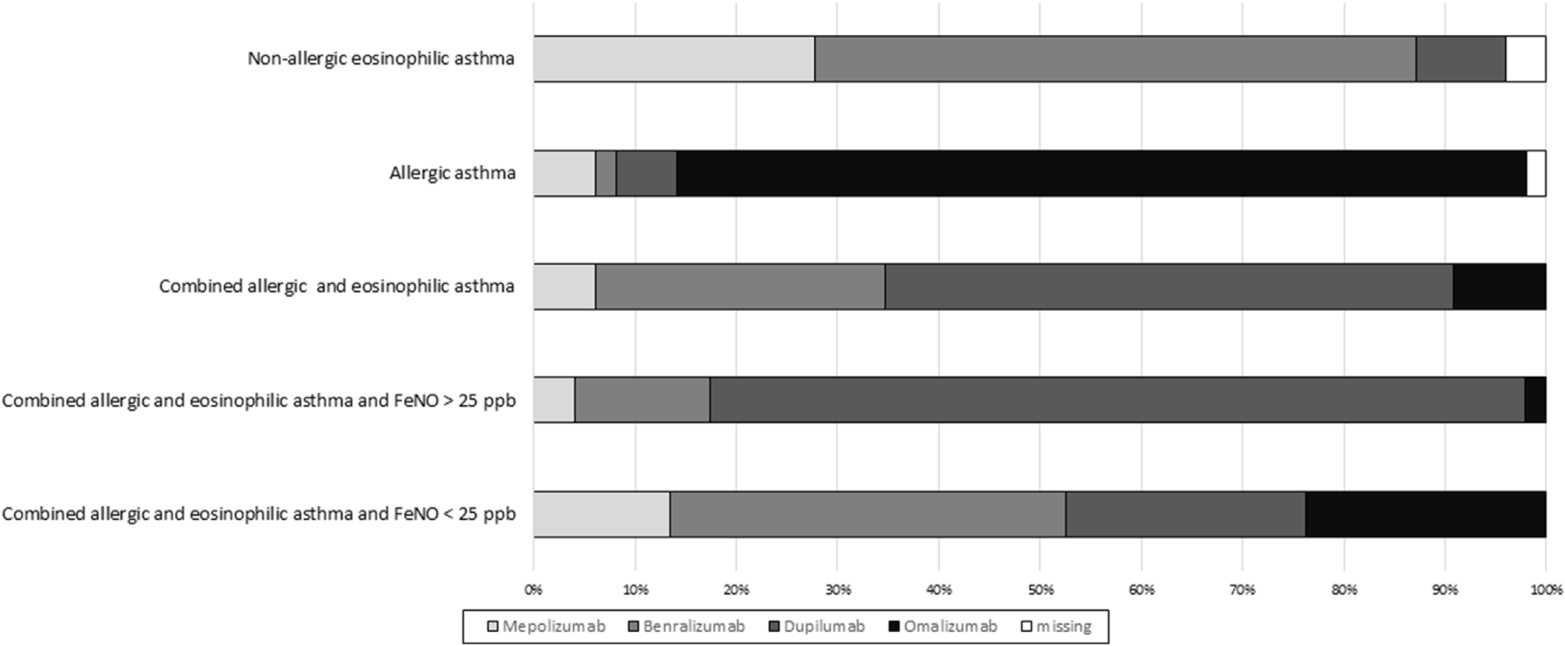
Initial choice of antibody for patients with severe uncontrolled asthma and certain features

Regarding the 3 biomarkers that were considered most important, participants recommended to measure FeNO (73%), and blood eosinophils (79%) at least twice, and total IgE *once* (91%). FeNO >25 ppb (66%) and blood eosinophils >300/μl (83%) were assumed as most relevant cut off-values. Seventy-two percent measured specific IgE before antibody therapy start. Most participants routinely assessed type-2 inflammation-related comorbidities including atopic dermatitis 98%; allergic rhinitis 94%, CRSwNP 94%, CRS 84%, CSU 72%, and food allergy 68%. For selection of biologic, CRSwNP and atopic dermatitis were the most important co-morbidities; whereas food-allergy scored the lowest interest. Next, we asked which antibodies the participants used initially according to phenotype and biomarkers (Fig. 3).

Additionally, we asked whether participants saw a difference between the effectiveness of mepolizumab and benralizumab: 47% thought that there is no difference; 89% regarded mepolizumab and benralizumab being equal in regard to safety. Most participants additionally thought that there was no difference in the effectiveness of both drugs on nasal polyps (81%). Comparing effectiveness on asthma outcome parameters of anti-IL5/IL5R. vs dupilumab, 26% of participants favor anti-IL5/IL5R and 19% favor dupilumab, while 53% did not observe differences. Concerning safety, 62% saw no difference, 4% considered dupilumab, and 30% anti-IL5/IL5R therapy as safer.

### Assessment of response to biologics

Seventy-two percent of pulmonologists assessed response to biologics for the first time 3–4 months after initiation and reassessed response every 3–4 months, whereas 23% used a 6 months interval. After the first year, 55% stuck to the 3–4 months interval while 34% used 6 months-intervals, others used individual intervals. For re-assessments, participants used exacerbation rate (96%), lung function (94%), OCS dose (94%), overall benefit (patients’ statement) (94%), and symptom scores (89%). Over 90% of participants used the Asthma Control Test (ACT) score for disease control assessment, while 13% used the Asthma Control Questionnaire 5 (ACQ5); only the ACT was considered “relevant”.

**Fig. 4:**
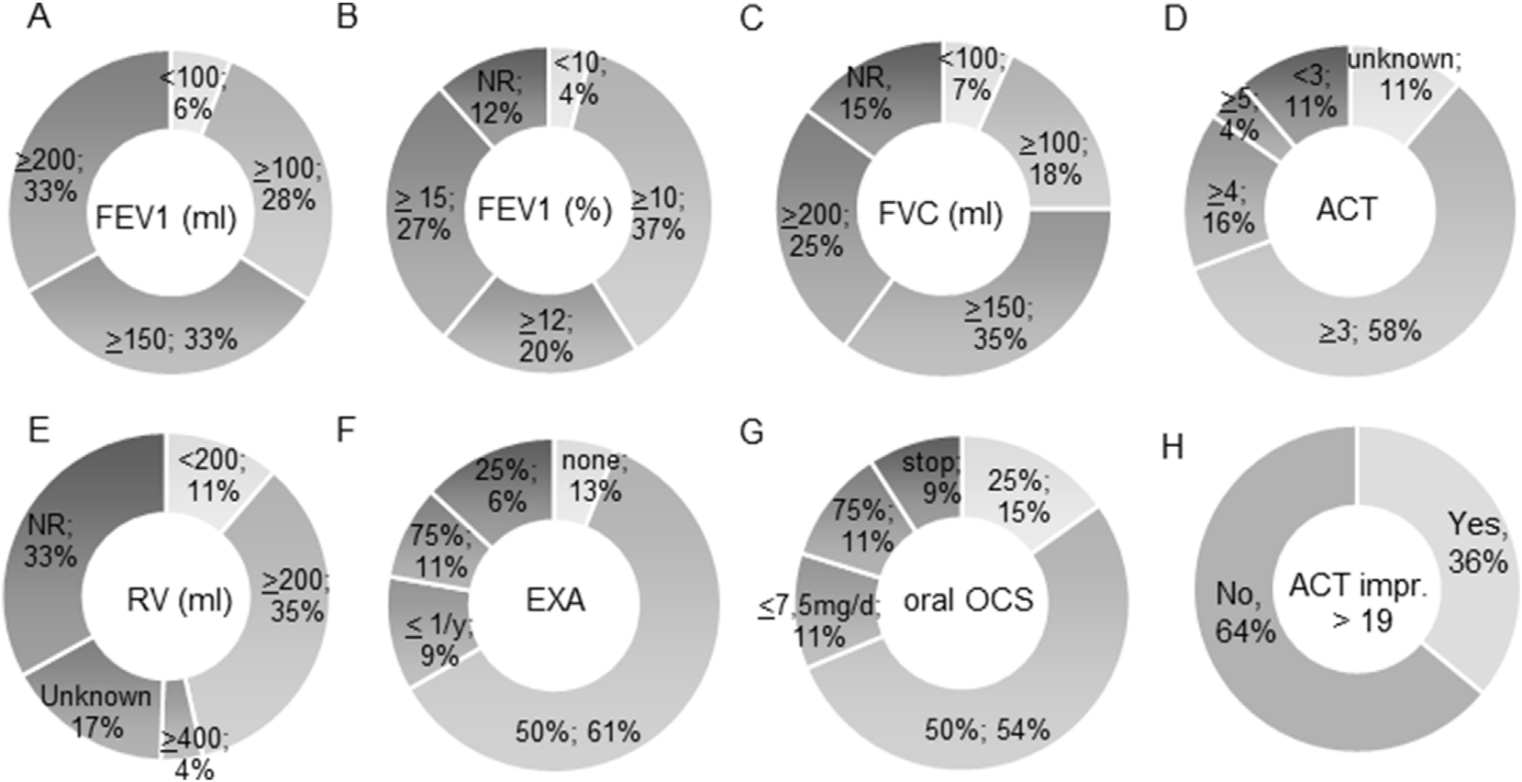
Participants' thresholds for clinically relevant differences for evaluation of treatment response – threshold for suggested improvement and increasement of (A) FEV1 (ml) or (B) FEV1 (%), (C) FVC (ml), (D) ACT and reduction of (E) RV (ml), (F) exacerbations (EXA) (%) and (G) oral steroids (OCS). Answer if absolute improvement of ACT of more or equal to 19 points is needed for treatment response (H)

Further, we explored the participants’ thresholds for clinically relevant differences for evaluation of treatment response (Fig. 4). The thresholds used for FEV1 ranged from <100 ml to <10% predicted to >200 ml and >15%. A proportion of participants did not consider FVC (15%) and RV (33%) relevant for response assessment, but the majority also considered these plethysmography parameters at varying thresholds. Regarding ACT score we asked if an improvement of ACT to an absolute value of above 19 points is a requirement to classify a patient as an antibody therapy responder: 64% of participants denied and 36% agreed.

Eighty-five percent of participants considered response of comorbidities to evaluate the treatment response. In regard to chronic rhinosinusitis with nasal polyps (CRSwNP) 70% used an overall statement of the patient to judge the effect, 62% consulted a Ear Nose and Throat (ENT) doctor and 28% used specific symptom scores (SNOT 22 or SCT) (multiple answers were possible). For atopic dermatitis, 55% of participants consulted a dermatologist and 23% used the overall statement of the patient (multiple answers were possible). A majority of 60% did not routinely assess exercise capacity by objective measurements, most often they used (47%) open questions, beside 6 min walk test (26%) or cardiopulmonary exercise testing (CPET) (30%). Seventy-five percent assessed quality of life mostly using open questions.

For stratification of treatment response 85% differentiated between “responder”, “partial responder”, and “non responder”, while 15% differentiated between “responder” and “non-responder”.

**Fig. 5:**
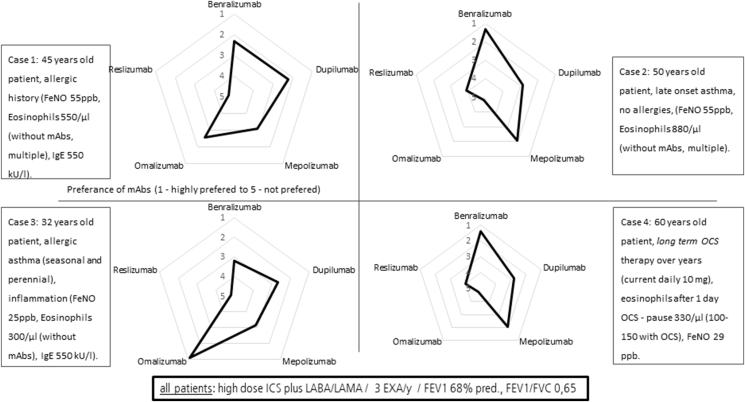
Choice of initial biologic in four example patients according to biomarkers and phenotypes. For all patients we assumed a background therapy of high dose ICS plus LABA/LAMA, 3 exacerbations in the last year, FEV1 = 68% pred. and FEV1/FVC 0,65

Further, we designed 4 typical patient examples with biomarker constellations to retrieve the choice of initial treatment (Fig. 5). Thirty-six percent of participants thought that more than 10–24 patients were needed to acquire sufficient experience to decide when to switch antibody therapy, 38% thought that 25–50 patients, and 17% thought that more than 50 patients were needed respectively.

### Treatment switch

When an insufficient response to the first antibody was found, participants chose an alternative biologic depending on comorbidities (CRSwNP was most relevant, followed by atopic dermatitis, chronic spontaneous urticaria and allergy). In case of insufficient response to anti-IL5 biologic, half of the participants used dupilumab and half the alternative anti-IL5/IL5R biologic. In case of treatment failure of omalizumab or reslizumab, the first choice was dupilumab followed by benralizumab. When dupilumab failed as initial biologic treatment, most participants chose benralizumab next.

## Discussion

The present study analyses current opinions and practice of initiation and choice, response assessment, and switch of biologic therapy in severe asthma among specialists in Germany.

We found that most participants phenotype their patients prior to biologic start using repetitive measurement of biomarkers (blood eosinophils, FeNO, IgE) and assessment of type 2-inflammation related comorbidities. Whereas GINA defines lower thresholds for type 2-inflammation biomarkers (blood eosinophils ≥150/μl, FeNO ≥20 ppb (both repeated up to 3x),^5^ sputum eosinophils ≥2%, and/or allergic asthma), specialists in Germany mostly use higher thresholds (blood eosinophils >300/μl and FeNO >25 ppb repeated twice), while sputum is not routinely used.

Co-morbidities played a major role for initial treatment choice. The participants saw a stronger effect of dupilumab than of anti-IL5/IL5R antibodies on CRSwNP. This is in line with real-life effectiveness reported recently.^6^ Response of CRSwNP in patients with severe asthma was compared using dupilumab or anti-IL5/IL5R therapy. Under dupilumab the reduction of nasal symptoms (visual analogue scale) was –3 vs. –1 under anti IL5/IL5R antibody.

Using example patients with typical clinical and biomarker constellations, we found different approaches with regard to choice of first biologic therapy. For non-allergic eosinophilic asthma, the majority (>85%) of participants chose anti-eosinophilic drugs first, with more often preferred benralizumab than mepolizumab, although there are no head-to-head studies to give an evidence-based guidance. In contrast, previous retrospective analysis found no differences in effectiveness between anti-IL5/IL5R therapy.^7^

For allergic asthma most participants (>80%) chose omalizumab while more than half of the participants chose dupilumab for mixed allergic and eosinophilic asthma. The choice of antibody in mixed allergic and eosinophilic asthma was also influenced by FeNO, with preference for dupilumab when FeNO was >25 ppb and preference of benralizumab when FeNO was <25 ppb.

In contrast to a previous study by Mattei et al^8^ that included 20 experts to define “failure criteria” for anti-IL5 therapy, our survey focused on response rather than non-response to all available biologics. Mattei et al summarized “failure” as: 1.) absence of a reduction in exacerbation rates by at least 25%, or 2.) absence of a reduction in oral corticosteroid therapy by at least 25% of the initial dosage, or 3.) the occurrence of emergency room visits or hospitalizations (with or without intensive care) after 6 months of treatment and suggested to stop treatment when these criteria are met to avoid unnecessary exposition of non responding patients.^9^ In the present study, 80% of participants re-assessed the antibody therapy using exacerbation rate, lung function, OCS dose, overall benefit as well as ACT score. As threshold for response, more than half of the participants thought that exacerbation rate and OCS dosage need to be reduced by at least 50%, representing a higher threshold but the same time period as agreed by Mattei et al. A six-month time point seems to be agreed for definite response assessment. For assessment of symptoms ACT test is the only questionnaire that is commonly used among pulmonologists in Germany. A majority considered an increase of 3 or more relevant, which corresponds to the minimal clinically important difference (MCID) of this test.^9^ However, there was no agreement among the participants regarding thresholds for response of pulmonary function parameters. This reflects the lack of established MCID for pulmonary function test (PFT) parameters in asthma. Great variation of response to biologics may be observed in clinical practice ranging from large improvement (eg, increase of FEV1 up to 1l,^10^ and normalization, to minimal or no change even in patients otherwise classified as responders). Interestingly, while all specialists use a threshold for FEV1 response, some specialists do not routinely use forced vital capacity (FVC) or residual volume (RV) for response assessment. Still, the majority of specialists takes into account FVC and RV, probably reflecting frequent use of plethysmography in care of patients with severe asthma in Germany.

In the multinational Core Outcome Measures sets for paediatric and adult Severe Asthma (COMSA) working group, that developed an outcome measurement set for severe asthma patients, different professional groups with different motives worked together.^11^ In our survey, only physicians who currently care for patients with severe asthma and prescribe biologics took part. However, COMSA proposed similar outcome measurement “sets” as used by physicians here. The final COMSA recommendation was to use Severe Asthma Questionnaire (SAQ), Asthma control questionnaire (ACQ-6 with symptoms and rescue medication use reported separately), FEV1, severe exacerbations, and oral OCS use.^12^ Use of ACQ-6 in COSMA probably reflects regional differences as the majority of participants from COSMA were from the United Kingdom, while participants from Germany in our study used ACT. Both symptom questionnaires use similar questions resulting in a similar classification of well-controlled, partly-controlled, and uncontrolled asthma, but the time-frame covered differs with 1 week for ACQ-6 and 4 weeks for ACT. Hence, both questionnaires are actually too short for assessment of response to biologics and would require multiple repetitions in between physician's visits to obtain a reliable measure for the whole time-period. Other questionnaires such as Asthma Impairment and Risk Questionnaire (AIRQ) have been developed to meet these issues,^13^ but are currently not widely used. The Seattle Angina Questionnaire (SAQ), a quality-of-life questionnaire, was also not used here, possibly reflecting reluctance to apply multiple questionnaires for a patient in daily practice. However, in regard to severe exacerbations and oral steroid use, participants already apply the COMSA recommendations in daily practice. While COMSA has not published thresholds and weighting of the recommended parameters yet, other scores have recently been proposed. The FEOS score (FEV1, Exacerbations, Oral corticosteroids, Symptoms) divides each parameter into 4–5 response classes and applies a weighted score for each parameter resulting in a fine classification of response. A recent proposal from Germany, the Biologic Asthma Response Score (BARS),^11^ also proposes use of symptoms, exacerbations, and oral steroid use, but in more simple classification in insufficient — intermediate — good response for each parameter as well as the combined score. It aims to provide a tool that is easily applicable in routine clinical practice. All these proposals still need validation in larger multinational cohorts, and as no recommendations regarding thresholds or scores have been made by guidelines, thresholds used by pulmonologists in Germany here show large variations.

The majority of participants distinguished between responder, partial responder, and non-responder underlining their differentiated perspective on this issue. Similarly, in the current German asthma guideline that was issued in March 2023 (thus after data acquisition for this project was finished) a ternary classification of super response, partial response, and non-response has been proposed.^14^ Interestingly, the most important factors to judge “response to biologics” were “overall benefit stated by the patient”, OCS dosage, exacerbations, ACT, and lung function (FEV1). CRSwNP was the co-morbidity with the highest impact on switch of antibody. Forty-five percent of participants declared that if they expected further improvement using another biologic, they would change therapy even in “responding” patients. Papaioannou et al already made suggestions for biologic switch^15^ based on biomarkers and patient characteristics. Following our data, the proposed use of sputum eosinophils does not reflect clinical practice of specialists in Germany.

A potential limitation of this study is that it reflects only the current situation in Germany depending on approval status and reimbursement of antibody therapies, which differ in other countries. On the other side it is a strength that in Germany all the investigated biologics were available and reimbursed by the statutory health insurance, so that no direct financial interests influence the prescription of the biologic. Consequently, results of the questionnaire mainly represent a patient centered decision-making process. A further limitation might be, that no question about skin prick test was formulated.

## Conclusion

This study showed general trends of pulmonologists current approach to use antibody therapies for severe asthma in Germany. With increasing treatment options available for patients with severe asthma, there is a need for more evidence and clear recommendations on choice, evaluation of response, and switching of biologics.

## Abbreviations

ACT, Asthma Control Test; AD, Atopic dermatitis; GINA, Global Initiative for Asthma; GAN, German Asthma Net; IQR, Interquartile range; ICS, Inhaled corticosteroids; LABA, Long acting beta agonists; LAMA, Long-Acting Muscarinic Antagonists; FeNO, Fractional exhaled nitric oxide; IgE, Immunglobulin E; mAbs, Monoclonal antibody; CRS, Chronic rhinosinusitis; CRSwNP, Chronic rhinosinusitis with nasal polyps; CSU, Chronic spontaneous urticaria; IL5, Interleukin 5; IL5R, Interleukin 5 receptor; FEV1, Forced expiratory volume in 1 s; FVC, Forced vital capacity; RV, Residual volume; OCS, Oral corticosteroids; EXA, Exacerbation; ENT, Ear Nose Throat; SNOT, Sino-Nasal Outcome Test; SCT, Sinus Control Test; CPET, Cardio pulmonary exercise testing

## Funding

None.

## Author contributions

Hendrik Suhling - significant contribution in conception, design, execution and interpretation.

Dirk Skowasch - significant contribution in execution and interpretation.

Karl-Christian Bergmann - significant contribution in execution and interpretation.

Carlo Mümmler - significant contribution in execution and interpretation.

Roland Buhl - significant contribution in execution and interpretation.

Rainer Ehmann - significant contribution in execution and interpretation.

Eckard Hamelmann - significant contribution in execution and interpretation.

Marco Idzko - significant contribution in execution and interpretation.

Margret Jandl - significant contribution in execution and interpretation.

Christian Schulz - significant contribution in execution and interpretation.

Olaf Schmidt - significant contribution in execution and interpretation.

Christian Taube - significant contribution in execution and interpretation.

Stephanie Korn - significant contribution in conception, design, execution and interpretation.

Katrin Milger - significant contribution in conception, design, execution and interpretation.

## Ethics statement

This is a survey study that involved voluntary participation.

## Declaration of competing interest

HS reports fees for lectures or consultations from Astrazeneca, GSK, Novartis, Sanofi all outside the submitted work.

DS reports fees for lectures or consultations from AstraZeneca, Bayer, Boehringer Ingelheim, Chiesi, GSK, Janssen, MSD, Sanofi, all outside the submitted work.

KCB reports speaker fees from AstraZeneca, GSK, Novartis, Sanofi all outside the submitted work.

CM no conflicts of interest.

RB reports consulting fees or honoraria for lectures from ALK, AstraZeneca, Berlin-Chemie, Boehringer-Ingelheim, Chiesi, Cipla, GSK, Novartis, Roche, Sanofi, and TEVA, and grants to Mainz University Hospital for research or clinical trials, or both from Boehringer Ingelheim, GSK, Novartis, and Roche.

RE no conflicts of interest.

EH is funded by the German Ministry of Education and Research (BMBF) (CHAMP, Project Number: 01GL1742D) for characterisation of children and adolescents with severe asthma. He reports personal fees from ALK, Boehringer Ingelheim, GSK, Leti Pharma, Novartis, Nutricia, Sanofi, and Stallergenes all outside the submitted work.

MI reports personal fees from AstraZeneca, Berlin-Chemie, Boehringer Ingelheim, Chiesi, CSL-Behring, Menarini, MSD, Novartis, Roche, Sanofi, all outside the submitted work.

MJ no conflicts of interest.

CS received personal fees from AstraZeneca, Boehringer Ingelheim, Novartis, and Sanofi, all outside the submitted work.

OS reports speaker fees from AstraZeneca, Novartis, Sanofi and Boehringer Ingelheim, all outside the submitted work.

CT no conflicts of interest.

KM reports speaker fees from AstraZeneca, GSK, Novartis, Sanofi, all outside the submitted work.

SK reports speaker fees from AstraZeneca, GSK, Novartis, Sanofi, all outside the submitted work.
