# Supplementary material for: Initiation, response assessment, and switch of antibody therapies in patients with severe asthma – A survey among German specialists
Source: World Allergy Organ J. 2023 Nov 15;16(12):100844. doi: 10.1016/j.waojou.2023.100844 (PMC10682826; doi:10.1016/j.waojou.2023.100844)
Supplement: Multimedia component 1 [file mmc1.pdf]

oFb - der onlineFragebogen

Dear collegues,

We ask you to complete the following questions for the Delphi – Consensus Project regarding antibody choice, antibody treatment and change of antibody therapy in the treatment of severe bronchial asthma.

1. How old are you? (years)

2. For how long have you treated patients with asthma? (years)

3. How many patients with severe asthma on antibody therapy do you currently treat?

4. Where do you practice?

Hospital

Private practice („Niederlassung")

other

Please answer the following questions about antibody treatment of patients with severe asthma

5. Which parameters do you use to categorize the patients as „severe asthma" eligible for antibody therapy? Please sort from the most to least relevant parameter.

**Exacerbations**	**Hospitalisations**	1
**Lung function**	**ACT / ACQ**	2
		3

Medication (ICS / LABA / LAMA)

Medication OCS

Days missed at work / school

4

5

6

7

6. Is it mandatory that the patient takes OCS currently or did so in the past 12 months to be eligible for antibody therapy? (at least during exacerbations)

Yes

No

Do not know

7. Which medication(s) must be used already for a patient to be eligible for antibody therapy? (Choose all that apply)

Moderate ICS

High dose ICS

LABA

LAMA

OCS

Montelukast

8. Do you consider a LAMA treatment attempt obligatory before antibody therapy?

Yes

No

Do not know

9. Which biomarker measurements do you consider essential before initiation of antibody therapy?

Blood eosinophils

Sputum eosinophils

FeNO

Total IgE

Specific IgE

Blood neutrophils

10. How many FeNO values do you consider necessary prior to antibody therapy?

0

1

2

>2

11. How many blood eosinophilic counts do you consider necessary prior to antibody therapy?

0

1

2

>2

12. How many IgE values do you consider necessary prior to antibody therapy?

0

1

2

>2

13. Do you decide which antibody you choose based on comorbidities?

Yes

No

Do not know

14. Optional: Which comorbidities do you routinely ask about / assess?

Allergic Rhinitis

Food Allergy

Chronic spontaneus urticaria

Chronic rhinosinusitis

Chronic rhinusinositis with nasal polyps

Atopic Dermatitis

15. Do you decide which antibody you choose based on the biomarkers?

Yes

No

16. Which antibody therapy would you prefer for severe non-allergic eosinophilic asthma?

Omalizumab

Mepolizumab

Reslizumab

Benralizumab

Dupilumab

17. Which antibody therapy do you prefer for severe allergic asthma?

Omalizumab

Mepolizumab

Reslizumab

Benralizumab

Dupilumab

18. Which antibody therapy do you prefer for severe combined allergic and eosinophilic severe asthma?

Omalizumab

Mepolizumab

Reslizumab

Benralizumab

Dupilumab

19. Which antibody therapy do you prefer for severe combined allergic and eosinophilic severe asthma and FeNO > 25 ppb

Omalizumab

Mepolizumab

Reslizumab

Benralizumab

Dupilumab

20. Which antibody therapy do you prefer for severe combined allergic and eosinophilic severe asthma and FeNO < 25 ppb

Omalizumab

Mepolizumab

Reslizumab

Benralizumab

Dupilumab

21. Which cut-off value do you use to define eosinophilic asthma ? (Eosinophils per µl)

>150

>300

>450

>600

22. Which cut-off value do you to use to define high-FeNO? (in ppb)

> 20

>25

>35

>50

23. Which co-morbidities do you consider in selecting your patient's antibody treatment ? (Please select in order of importance)

Urticaria

Atopic dermatitis

CRSwNP

CRSsNP

1

2

3

Food allergies

4
5

24. Do you see differences between Mepolizumab and Benralizumab regarding treatment efficacy ?

No

Yes – Benralizumab more effective

Yes – Mepolizumab more effective

25. Do you see differences between Mepolizumab and Benralizumab regarding safety?

No

Yes – Benralizumab safer

Yes – Mepolizumab safer

26. Do you see differences between Mepolizumab and Benralizumab regarding efficacy on CRSwNP?

No

Yes – Benralizumab more effective

Yes – Mepolizumab more effective

27. Do you see differences between Anti-IL-5 biologics and Dupilumab regarding efficacy in asthma outcome parameters?

No

Yes – Dupilumab more effective

Yes – Anti-IL5/ IL5R more effective

28. Do you see differences between Anti-IL-5 biologics and Dupilumab regarding safety?

No

Yes – Dupilumab safer

Yes – Anti IL5 / IL5R safer

29. Do you see differences between Anti-IL-5 biologics and Dupilumab regarding efficacy on CRSwNP?

No

Yes – Dupilumab more effective

Yes – Anti IL5 / IL5R more effective

In the next section, questions about response to antibody treatment follow.

30. In your oppinion, what is the best time point to assess treatment response for the FIRST time?

3- 4 months

6 months

12months

Later

31. How often do you reassess the response of an ongoing long-term antibody therapy (>12 months)?

Every 3 – 4 months

Every 6 months

Every 12 months

No fixed interval

Individual choice

32. Which factors do you routinely obtain to assess response to antibody therapy? (select all that apply)

Overall benefit stated by the patient

OCS dosage

Number of exacerbations

Symptom score

Lung function

Quality of life

Physical Activity

Work / school day missing

Blood eosinophil levels (decline)

FeNO levels (decline)

Change/deescalation of co-treatments e.g. theophylline, LTRA

33. Which parameters are most important for response assessment? Please order from most important (1) to least important (11).

**Overall benefit stated by the patient**	**OCS dosage**	1
	**Number of exacerbations**	2
**Symptom score**	**Lung function**	3
**Quality of life**	**Physical Activity**	4
**Work / school days missed**	**Blood eosinophil levels (decline)**	5
**FeNO levels (decline)**	**Change/deescalation of co-treatments e.g. theophylline, LTRA**	6
		7
		8
		9
		10
		11

34. Which symptoms scores do you routinely use to assess treatment response regarding asthma control? (select all that apply)

ACT

ACQ5

ACQ6

ACQ7

none of these

35. Which of these symptom scores do you consider most important?

ACT

ACQ5

ACQ6

ACQ7

none of these

36. Which of these lung function parameters do you consider important to evaluate treatment response regarding lung function? (select all that apply)

FEV1

FVC

FEV1/FVC

MEF

RV

RV/TLC

Raw

37. Which of these lung function parameters do you consider most important in this assessment? (Please sort from most important (1) to least important(7))

**FEV1**	**FVC**		**1**
**FEV1/FVC**	**MEF**		**2**
**RV**	**RV/TLC**		**3**
**Raw**			**4**
			**5**
			**6**
			**7**

38. Which is the smallest FEV1 change that you consider a clinically relevant treatment response? (absolute values in ml)

<100

>/= 100

>/= 150

>/= 200

39. Which is the smallest FEV1 change that you consider a clinically relevant treatment response? (relative values in % improvment)

<10

>/= 10

>/= 12

>/= 15

I do not know

I do not consider this parameter „relevant“

40. Which is the smallest FVC change that you consider a clinically relevant treatment response? (absolute values in ml)

<100

>/= 100

>/= 150

>/= 200

I do not know

I do not consider this parameter „relevant“

41. Which is the smallest RV change that you consider a clinicaly relevant treatment response? (absolute values in ml)

<200

>/= 200

>/= 400

I do not know

I do not consider this parameter „relevant“

42. Which is the smallest ACT change that you consider a clinicaly relevant treatment response?

<3

>/= 3

>/= 4

>/= 5

I do not know

43. Do you consider an absolute improvement of ACT above 19 points a requirement to classify a patient as a antibody therapy responder?

Yes

No

I do not know

44. Which is the minimum reduction of OCS-courses due to exacerbations that you consider clinically relevant“?

At least 25% reduction

At least 50% reduction

At least 75% reduction

Patient must achieve absolute number of 1 or less

Patient must be exacerbation-free

45. In patients with long term OCS therapy:

Which is the minimum dose reduction necessary to consider the patient a „treatment responder“?

At least 25% reduction

At least 50% reduction

At least 75% reduction

Patient must achieve an absolute dosage <7.5mg/d

Patient must achieve complete stop of OCS

46. Do you use individualised „marker“ symptoms to judge antibody response?

Yes

No

47. Optinal: Which individualised marker do you use ?

48. Do you consider changes in asthma comorbidities to asses antibody treatment response?

Yes

No

49. If you consider changes in Chronic Rhino-Sinusitis with or without nasal polyps (CRSw/sNP) to evaluate antibody treatment response, how do you assess these changes? (Choose all that apply)

Overall statement by the patient

Visual analogue scale (VAS)

Symptom score (e.g. SNOT-22 or SCT)

Interdisciplinary consultation of an ENT doctor

50. If you consider changes in Atopic Dermatitis to evaluate antibody treatment response, how do you assess these changes? (Choose all that apply)

Overall statement by the patient

Visual analogue scale (VAS)

Symptom score

Interdisciplinary consultation of an dermatologist

51. Do you routinely assess exercise capacity in patient with severe asthma (either through questionnaire or by exercise test)?

Yes

No

52. Optional: How do you assess exercise capacity?

MRC questionnaire

Open question

Other questionnaire

6min walk test

Cardio-pulmonary exercise testing

Activity Tracker

53. Do you routinely assess quality of life in patients with severe asthma ?

Yes

No

54. Optional: How do you assess quality of life?

Open question

AQLQ

SGRQ

Visual Analogue Scale

55. How do you stratify antibody treatment response?

Responder / partial responder / non responder

Responder and non responder

In the next section, questions about change of antibody treatment follow.

56. If a patient initially treated with mepolizumab does not respond to therapy, what would be your primary antibody choice for a subsequent antibody therapy? (Assuming that the patient is eligible for all these antibodies).

Benralizumab

Dupilumab

Omalizumab

Reslizumab

57. If a patient initially treated with reslizumab does not respond to therapy, what would be your primary antibody choice for a subsequent antibody therapy? (Assuming that the patient is eligible for all these antibodies).

Mepolizumab

Dupilumab

Omalizumab

Benralizumab

58. If a patient initially treated with dupilumab does not respond to therapy, what would be your primary antibody choice for a subsequent antibody therapy? (Assuming that the patient is eligible for all these antibodies).

Mepolizumab

Benralizumab

Omalizumab

Reslizumab

59. If a patient initially treated with omalizumab does not respond to therapy, what would be your primary antibody choice for a subsequent antibody therapy? (Assuming that the patient is eligible for all these antibodies).

Mepolizumab

Benralizumab

Dupilumab

Reslizumab

60. Which comorbidities do you consider for the choice of an alternative antibody upon failure to respond to an initial antibody therapy? Please sort by relevance from high (1) to low (7)

**Urticaria**	**Atopic dermatitis**	**1**
**CRSwNP**	**CRSsNP**	**2**
**Food allergies**	**Seasonal allergies**	**3**
**Perennial allergies**		**4**
		**5**
		**6**
		**7**

61. Would you consider a change in antibody therapy despite clinical improvement because you expect more positive effects on a different antibody therapy?

No   Yes

62. How many asthma patients do you think a pneumologist has to have treated with antibody therapy to have aquired sufficient experience to decide when to switch antibody therapy?

> 50	10 to 24
25 to 50	< 10

For the following questions, we would like you to consider some case examples:

For all cases, the patients clinical situation and history are as follows: A therapy with high dose ICS plus LABA / LAMA is given, patients had 3 exacerbations prior antibody therapy, no OCS long term therapy (except case 4), a FEV1 of 68% predicted, FEV1/FVC 0,65.

63. Case 1: 45 years old patient, allergic history (FeNO 55ppb, Eosinophils 550/µl (without mbs, multiple), IgE 550 kU/l).

Please sort your choice of antibody from most appropriate (1) to least appropriate (5).

Benralizumab **Dupilumab**

Mepolizumab **Omalizumab**

Reslizumab

1
2
3
4
5

64. Case 2: 50 years old patient, late onset asthma, no allergies, (FeNO 55ppb, Eosinophils 880/µl (without mbs, multiple).

Please sort your choice of antibody from most appropriate (1) to least appropriate (5).

Benralizumab **Dupilumab**

Omalizumab **Mepolizumab**

Reslizumab

1
2
3
4
5

65. Case 3: 32 years old patient, allergic asthma (seasonal and perennial), inflammation (FeNO 25ppb, Eosinophils 300/µl (without mbs), IgE 550 kU/l).

Please sort your choice of antibody from most appropriate (1) to least appropriate (5).

Benralizumab | **Dupilumab** | **Omalizumab** | **Mepolizumab** | **Reslizumab**

1
2
3
4
5

66. Case 4: 60 years old patient, long term OCS therapy over years (actual 10 mg), eosinophils after 1 day OCS – pause 330/µl (100-150 with OCS), FeNO 29 ppb.

Please sort your choice of antibody from most appropriate (1) to least appropriate (5).

Benralizumab | **Dupilumab** | **Omalizumab** | **Mepolizumab** | **Reslizumab**

1
2
3
4
5

Weiter

0% ausgefüllt
